# Diverse Morbidity and Mortality Among Infants Treated with Venoarterial Extracorporeal Membrane Oxygenation

**DOI:** 10.7759/cureus.263

**Published:** 2015-04-07

**Authors:** Sigrid Bairdain, Peter Betit, Nancy Craig, Kimberlee Gauvreau, Peter Rycus, Jay M Wilson, Ravi Thiagarajan

**Affiliations:** 1 Department of Surgery, Boston Children's Hospital; 2 Department of Respiratory Therapy, Boston Children's Hospital; 3 Department of Cardiology, Boston Children's Hospital; 4 ELSO Executive administrator, ELSO; 5 Department of Pediatric Surgery, Boston Children's Hospital

**Keywords:** venoarterial extracorporeal membrane oxygenation (va-ecmo), infants, mortality, morbidity

## Abstract

Background: Venoarterial extracorporeal membrane oxygenation (VA-ECMO) is utilized for cardiopulmonary failure. We aimed to qualify and quantify the predictors of morbidity and mortality in infants requiring VA-ECMO.

Methods: Data was collected from 170 centers participating in the extracorporeal life support organization (ELSO) registry. Relationships between in-hospital mortality and risk factors were assessed using logistic regression. Survival was defined as being discharged from the hospital.

Results: Six hundred and sixty-two eligible records were reviewed. Mortality occurred in 303 (46%) infants. Congenital diaphragmatic hernia patients (OR=3.83, 95% CI 1.96-7.49, p<0.001), cardiac failure with associated shock (OR= 2.90, 95% CI 1.46-5.77, p=0.002), and pulmonary failure including respiratory distress syndrome (OR=4.06, 95% CI 1.72-9.58, p=0.001) had the highest odds of mortality in this cohort. Birth weight (BW) < 3 kg (OR=1.83, 95% CI 1.21-2.78, p=0.004), E-CPR (OR=3.35, 95% CI 1.57-7.15, p=0.002), hemofiltration (OR=2.04, 95% CI 1.32-3.16, p=0.001), and dialysis (OR=6.13, 95% CI 1.70-22.1, p<0.001) were all independent predictors of mortality.

Conclusion: Infants requiring VA-ECMO experience diverse sequelae and their mortality are high.

## Introduction

Extracorporeal membrane oxygenation (ECMO) may be a life-saving modality for pediatric patients with either severe cardiac disease or respiratory failure [1]. However, these patients continue to represent a potentially very critical sub-cohort of intensive care unit patients, and their overall survival is still very poor [2]. According to the Extracorporeal Membrane Support Registry Report from 2012, approximately 13,000 patients have been treated with survival to discharge rates of 40%, 49%, and 39% for neonates, pediatric, and adults, respectively [2].

Since its inception, extracorporeal life support has been used as a modified form of cardiopulmonary bypass and has become an accepted therapeutic modality despite an ever increasing severely-ill cohort of patients [3]. Venoarterial extracorporeal membrane oxygenation (VA-ECMO) is a specific type of extracorporeal membrane life support. VA-ECMO differs from venovenous extracorporeal membrane oxygenation (VV-ECMO), another form of extracorporeal membrane life support, as VA-ECMO is utilized specifically for both hemodynamic and respiratory support; VV-ECMO is utilized primarily for respiratory support [4]. Physiological effects of VA-ECMO differ from VV-ECMO as VA-ECMO results in a mixture of pulsatile and non-pulsatile flow to multiple organ systems, require anticoagulation, and cannot be maintained indefinitely [4-5]. 

As aforementioned, different underlying etiologies require the use of VA-ECMO; however, common morbidities associated with VA-ECMO range from primary mechanical, renal, lung, and neurological, as well as neurodevelopment concerns [6-7]. There are numerous reports suggesting that underlying etiology, factors preceding, as well as congruent with the initiation of VA-ECMO are important predictors of morbidity and mortality. In particular, more recent reports by Zwiers, et al. (2012) and Askenazi, et al. (2011) have paid special attention to the relationship between the role of fluid overload and acute kidney injury as independent predictors of morbidity and mortality in cohorts of patients primarily on VA-ECMO [8-9]. Therefore, predicting survivability in this cohort, while weighing the constant morbidity and mortality risk in these complex patients, is a constant challenge for the treatment team. Using the extracorporeal life support organization (ELSO) registry data for a one-year time period, the aim of this study was to quantify and qualify the morbidity and mortality of a cohort of infants ages 0-1 years requiring VA-ECMO.

## Materials and methods

### Population

Following IRB approval (IRB #00010390) from the Boston Children's Institutional Review Board, infants aged 0-1 years that required VA-ECMO for a one-year period were retrospectively reviewed from the Extracorporeal Life Support Organization (ELSO) registry. The ELSO registry collects data on ECMO procedures performed and is prospectively collected from patients admitted to over 170 centers participating in the registry. The centers included are voluntary members. The ELSO dataset is based off either ICD-9 codes and/or CPT codes for procedures. It does not include specific dates, indications for initiation of specific therapies, or relationship to surgeries.

Criteria to initiate VA-ECMO and placement of cannulas (centrally or peripherally) varied based on center criteria and were at the discretion of the intensive care practitioner and surgeon. Subjects were excluded from the study if their form of support was other than VA-ECMO and only included those patients on VA-ECMO. In those patients that required more than one ECMO run, runs were combined. The ELSO database provided a patient identification (ID) and a run ID; the run IDs for the individual patient who had more than one run were combined and results were reported collectively on the individual patient.

### Definitions for underlying etiologies

The primary indication for VA-ECMO was reviewed by a pediatric cardiologist (RRT) and a critical care fellow (SB) and included: 1) pulmonary; 2) cardiac; and 3) emergency ECMO extracorporeal cardiopulmonary resuscitation (ECPR). Etiologies were further subdivided into the following categories: 1) acyanotic cardiac lesions; 2) cyanotic cardiac lesions; 3) pulmonary structural lesions, including congenital diaphragmatic hernia; 4) pulmonary lesions including persistent pulmonary hypertension of the newborn (PPHN); 5) cardiac failure and/or shock; 6) pulmonary failure including respiratory distress syndrome; 7) infectious etiologies; 8) pulmonary lesions resulting from aspiration; 9) other cardiac lesions; 10) other pulmonary lesions; and 11) lesions otherwise not categorized.

Acyanotic and cyanotic congenital cardiac heart disease (CHD) diagnoses were grouped and coded. Each patient was reviewed by a pediatric cardiologist (RRT) to ensure appropriate coding. These diagnoses were then sorted into one of three groups according to their dominant effect on cardiovascular physiology. Category 1 (acyanotic) included defects that primarily compromised systemic output (critical aortic stenosis, coarctation of the aorta, hypoplastic left heart syndrome (HLHS), and interrupted aortic arch). Category 2 was comprised of defects that create significant and sustained cyanosis (transposition of the great vessels, tetralogy of Fallot, critical pulmonary stenosis, pulmonary atresia, tricuspid atresia, and total anomalous pulmonary venous return). For those with dual diagnoses that would place them in multiple categories, a hierarchy was used to sort them into the most appropriate category based on physiology.

### Definitions for variables

Demographic, pre-ECMO support type, ECMO procedures and complications, patient complications, and outcomes were analyzed. Given the numerous pre-ECMO support types, these were reviewed by a pediatric cardiologist (RRT) and critical care fellow (SB) and were grouped into 5 categories. Category 1 contained pulmonary vasodilators and included inhaled nitric oxide (iNo) and sildenafil. Category 2 contained medications whose mechanism of action was characterized by inotropic support; these included dopamine, dobutamine, vasopressin, epinephrine, and norepinephrine. Category 3 was characterized as adjuncts to care and included the following: tris(hydroxymethyl) aminomethane (THAM), bicarbonate, systemic hypothermia, systemic steroids, inhaled anesthetics, narcotics, paralysis, plasmapheresis, and abdominal compression. Category 4 was characterized by additional ventilator support and included the use of high-frequency oscillatory ventilation (HFOV), the use of surfactant and hyperventilation. Category 5 was characterized by additional cardiac support and included such entities as bypass, the use of a pacemaker or intra-aortic balloon, and the use of the Berlin Heart.

Complications were delineated by coding from the ELSO registry and were divided into broad and specific complications. Broad categories included complications affecting these systems: neurological, pulmonary, cardiac, renal, hemorrhagic, infectious, metabolic, and complications related to mechanical issues and specific pumps used. A full listing of all the individual complications with associated ELSO codes is seen in an appendix table. 

### Statistical analysis

Patient and procedural characteristics, pre-ECMO support variables, and overall complications were compared with infants who died in-hospital versus those who did not. Categorical variables were summarized as a number (percent) and compared using Fisher’s exact test; continuous variables were summarized as median (range) and compared with the Wilcoxon rank sum test. Variables significant at the 0.10 level in univariate analysis were considered for inclusion in a multivariable logistic regression model; p < 0.05 was required for inclusion in the final model. Analyses were performed using Stata 12.1 (StataCorp, College Station, TX).

## Results

### Demographics

Records of 662 eligible patients were reviewed. Males comprised approximately 55% percent of this patient population versus approximately 45% females. There was a distribution of the ages of the eligible patients; 465 (70%) were neonates or persons less than the age of 30 days, whereas 197 (30%) were infants, or those patients who were older than 30 days. The median age at cannulation was seven days (range: 0 days-365 days) and was not significantly different between the groups. Median weight at cannulation of those who died was 3.3 kg (range: 1.5-10.0 kg) versus 3.5 (range: 1.9-10.9 kg) who survived to discharge (p < 0.001). Three hundred and seventy-nine (57%) patients were cannulated via a carotid artery and internal jugular vein approach. Of the 662 eligible patients, 18 (3%) patients had two runs while four patients had three (1%) runs.

**Table 1:** Baseline characteristics of the cohort Kg: kilograms; Support type: Reason for initiation of support; VA-ECMO: Venoarterial extracorporeal membrane oxygenation; E-CPR/ECLS: emergency cardiopulmonary

In-Hospital Mortality	Total	Yes	No	P-Value
Characteristics		n = 303 (%)	n = 359 (%)	
Sex				0.53
Male	368 (56)	166 (55)	202 (58)	
Female	285 (43)	136 (45)	149 (42)	
Weight	3.3	3.2	3.5	<0.001
Information on procedures				
Support type				<0.001
Pulmonary	293 (44)	114 (38)	179 (50)	
Cardiac	278 (42)	134 (44)	144 (40)	
ECPR	91 (14)	55 (18)	36 (10)	
Hours on ECMO (n=659)	132	162	118	0.002
Any procedure pre-ECLS	375 (57)	177 (58)	198 (55)	0.43
Any procedure during ECLS	144 (22)	72 (24)	72 (20)	0.26
Any procedure post-ECLS	38 (6)	11 (4)	27 (8)	0.04
Any cardiac procedure pre-ECLS	155 (23)	72 (24)	83 (23)	0.85
Any cardiac procedure during ECLS	36 (5)	17 (6)	19 (5)	0.87
Any cardiac procedure post-ECLS	10 (2)	4 (1)	6 (2)	0.76
Hours admission to ECMO (n=636)	41	59	29	0.01
Hours intubation to ECMO (n=625)	26	26	26	0.59

### Univariate analysis

Information on ECMO, the influence of duration of ECMO, as well as the influence of any procedures performed in relation to ECLS, was also seen in Table 1. There was a significant difference in ECMO support type (p <0.001) at baseline. There was a predominance of cardiac ECMO support type in those who died versus those who survived; in those that survived, there was a predominance of pulmonary ECMO support type. Those who spent a longer median time on ECMO had a higher associated mortality (p < 0.002). There were no statistically significant differences found in regards to procedures on ECLS or concerning cardiac procedures. 

Pre-ECMO support also influenced in-hospital mortality. The use of pulmonary vasodilators including iNO and sildenafil, as well as additional ventilator support in the forms of HFOV, surfactant use, and a degree of hyperventilation appeared to be protective. For example, 63% of those who survived utilized pulmonary vasodilators, whereas only 50% of those who had died had been exposed to vasodilators as part of their pre-ECMO support regimen (p <0.001). Forty-three percent of those who survived utilized additional pulmonary regimens, although only approximately 30% of those who died had initiated HFOV and/or surfactant (p < 0.001). Additional adjuncts, inotropes, and additional cardiac support did not confer significantly increased odds of mortality in this cohort.  

**Table 2:** Pre-VA-ECMO support characteristics and mortality

In-Hospital Mortality	Yes N=303 (%)	No N=359 (%)	P-Value
Pre-VA ECMO support			
Pulmonary vasodilators	150 (50)	226 (63)	<0.001
Inotropic support	256 (84)	314 (87)	0.31
Adjuncts	230 (76)	273 (76)	1.0
Additional ventilator support	90 (30)	156 (43)	<0.001
Additional cardiac support	128 (42)	131 (36)	0.15

In-hospital mortality occurred in 303 (46%) of this cohort of patients. The influence of complications, associated morbidities, and in-hospital mortality is seen in Table 3. Ninety-two percent of those who died had experienced a complication (p<0.001). Complications ranged from neurological, pulmonary, cardiac, renal, hemorrhagic, infectious, metabolic, and complications related to mechanical issues. Neurological complications in the entire cohort included 66 (10%) hemorrhagic cerebral vascular accidents, 32 (5%) ischemic cerebral strokes, and brain death in 10 (2%) patients. The most common hemorrhagic complications included surgical site bleeding in 116 (18%) patients, cannula site bleeding in 82 (12%) patients, and disseminated intravascular coagulopathy in 42 (6%) patients. Gastrointestinal (GI) hemorrhage occurred in 12 (2%) patients. Hemofiltration, as part of the management schema, was also more prevalent in those who died than those who survived (p <0.001) as seen in Table 3. Renal complications, as defined by a serum creatinine level, included 34 (5%) patients with a serum creatinine (sCr) of between 1.5-3.0 mg/dl versus 18 (3%) patients with a sCr of > 3.0 mg/dl. The majority of cardiac complications in the entire cohort included persistent hypertension requiring medications in 80 (12%) patients, whereas pulmonary complications were evenly distributed between pneumothorax requiring treatment in 33 (5%) patients and pulmonary hemorrhage in 35 (5%) patients. Infection complications included 38 (6%) culture-proven infections while a white blood count (WBC) of less than 1,500 was seen in three (<1%) patients. Metabolic complications included primarily hyperglycemia, as pre-defined by a serum blood glucose of x > 240 mg/dl in 56 (8%) of the patient population. 

**Table 3:** Complications and in-hospital mortality

In-Hospital Mortality	Yes n=303 (%)	No n=359 (%)	P-Value
Complications			
Any complication	279 (92)	274 (76)	<0.001
Mechanical	117 (39)	106 (30)	0.02
Hemorrhagic	143 (47)	103 (29)	<0.001
Neurological	92 (30)	43 (12)	<0.001
Cardiac	191 (63)	173 (48)	<0.001
Pulmonary	42 (14)	21 (6)	<0.001
Infections	27 9)	13 (4)	0.005
Metabolic	75 (25)	63 (18)	0.03
Non-hemofiltration renal complication	50 (17)	20 (6)	<0.001
Serum creatinine			<0.001
<1.5	266 (88)	345 (96)	
1.5-3.0	28 (9)	5 (1)	
>3.0	9 (3)	9 (3)	
Serum creatinine > 1.5	37 (12)	14 (4)	<0.001
Hemofiltration	91 (30)	58 (16)	<0.001
Dialysis	23 (8)	3 (1)	<0.001

Mechanical complications were seen in 223 (34%) patients within this entire cohort. The most common mechanical complications included clots seen in the oxygenator (n=111/662, 17%), as well as mechanical failure (n= 31/662, 5%) of the entire cohort. There was not an increased mortality seen in comparing one pump versus the other in this patient cohort; however, the centrifugal pumps appeared to have a slightly higher rate of hemorrhagic hemolysis on univariate analysis (13% versus 7%, p =0.02). A listing of all respective ELSO complication codes is included. 

**Table 4:** ELSO codes for complications

Complication	ELSO Codes
Neurological	Neurologic: Brain death clinically determined (301)
	Neurologic: Seizures: clinically determined (311)
	Neurologic: Seizures: EEG determined (312)
	Neurologic: CNS infarction by US/CT (321)
	Neurologic: CNS hemorrhage by US/CT (322)
Mechanical	Mechanical: Oxygenator failure (101)
	Mechanical: Other tubing rupture (103)
	Mechanical: Pump malfunction (104)
	Mechanical: Clots: oxygenator (111)
	Mechanical: Clots: bridge (112)
	Mechanical: Clots: bladder (113)
	Mechanical: Clots: hemofilter (114)
	Mechanical: Clots: other (115)
	Mechanical: Air in circuit (121)
	Mechanical: Cracks in pigtail connectors (122)
	Mechanical: Cannula problems (131)
Hemorrhagic	Hemorrhagic: GI hemorrhage (201)
	Hemorrhagic: Cannulation site bleeding (202)
	Hemorrhagic: Surgical site bleeding (203)
	Hemorrhagic: Hemolysis (HGB > 50 mg/dl) (211)
	Hemorrhagic: Disseminated intravascular coagulation (DIC) (221)
Cardiac	Cardiovascular: Inotropes on ECLS (501)
	Cardiovascular: CPR required (502)
	Cardiovascular: Myocardial stun by echo (503)
	Cardiovascular: Cardiac arrhythmia (504)
	Cardiovascular: Hypertension requiring vasodilators (514)
	Cardiovascular: Tamponade: blood (541)
	Cardiovascular: Tamponade: serous (542)
	Cardiovascular: Tamponade: air (543)
Pulmonary	Pulmonary: Pneumothorax requiring treatment (601)
	Pulmonary: Pulmonary hemorrhage (602)
Renal	Renal: Creatinine 1.5 - 3.0 (401)
	Renal: Creatinine > 3.0 (402)
	Renal: Dialysis required (411)
	Renal: Hemofiltration required (412)
	Renal: CAVHD required (414)
Infectious	Infectious: Culture proven infection (701)
	Infectious: WBC < 1,500 (711)
Metabolic	Metabolic: Glucose < 40 (801)
	Metabolic: Glucose > 240 (802)
	Metabolic: Hyperbilirubinemia (> 2 direct or > 15 total) (821)

### Multivariate analysis

Congenital diaphragmatic hernia (CDH) (OR=3.83, 95% CI 1.96-7.49, p<0.001), cardiac failure with associated shock (OR= 2.90, 95% CI 1.46-5.77, p=0.002), and pulmonary failure, including respiratory distress syndrome (OR=4.06, 95% CI 1.72-9.58, p=0.001) patients had the highest odds of in-hospital mortality. Birth weight of < 3 kg (OR=1.83, 95% CI 1.21-2.78, p=0.004), E-CPR/extracorporeal life support (ELS) (OR=3.35, 95% CI 1.57-7.15, p=0.002), hemofiltration (OR=2.04, 95% CI 1.32-3.16, p=0.001), and dialysis (OR=6.13, 95% CI 1.70-22.1, p<0.001) were also all independent predictors of mortality. The presence of either pulmonary and/or neurological complications remained significantly associated with mortality; however, hemorrhagic, metabolic, and infectious complications, as well as type of pump, were no longer significant on multivariate analysis. Receiver operating curve (ROC) was 0.778 for the full model.  

**Table 5:** Multivariate analyses and in-hospital mortality Kg: kilograms; Support type: Reason for initiation of support; VA-ECMO: Venoarterial extracorporeal membrane oxygenation; E-CPR/ECLS: emergency cardiopulmonary; CDH: Congenital Diaphragmatic Hernia

Clinical Variables	Odds Ratio	95% CI	P-Value
Weight < 3 kg	1.83	(1.21, 2.78)	0.004
Support type			
Pulmonary	1.00	--	--
Cardiac	2.27	(1.21, 4.27)	0.01
E-CPR	3.35	(1.57, 7.15)	0.002
Hour on ECMO (↑ 10 hr.)	1.02	(1.01, 1.04)	<0.001
Pulmonary vasodilators pre-ECMO	0.55	(0.36, 0.85)	0.007
Any hemorrhagic complication	1.45	(0.99, 2.13)	0.05
Any neurological complication	2.81	(1.77, 4.45)	<0.001
Any pulmonary complication	2.52	(1.27, 4.99)	0.008
Hemofiltration	2.04	(1.32, 3.16)	0.001
Dialysis	6.13	(1.70, 22.1)	0.006
Diagnosis			
Cyanotic	1.68	(1.01, 2.80)	0.05
CDH/diaphragm	3.83	(1.96, 7.49)	<0.001
Cardiac failure	2.90	(1.46, 5.77)	0.002
Pulmonary failure	4.06	(1.72, 9.58)	0.001
Infectious	2.91	(1.32, 6.40)	0.008
Other	1.00	--	--

## Discussion

During this one-year study period, we abstracted data from the ELSO registry, which included data on ECMO procedures performed on 662 eligible patients. This is collected from patients admitted to over 170 centers participating voluntarily in the registry and allows for the recording of factors predicting morbidity and mortality. Although the ELSO registry is a robust tool for the investigation of ECMO, its use does have some limitations and data cannot be construed to suggest causality. The ELSO dataset does not include specific dates for interventions and/or indications for therapy; however, given the relative sample size, these data show a robust and wide array of morbidity and mortality experienced by this particular cohort of infants on VA-ECMO.

Patient selection, timing of the application of ECMO and mode of ECMO continue to be important factors in predicting eventual outcomes in these cohorts of patients [10]. VA-ECMO, unlike VV- ECMO, results in mixture of pulsatile and non-pulsatile blood flow and may exquisitely alter blood flow patterns to the cerebrum, pulmonary vasculature, and renal vasculature; not appreciated as much in VV-ECMO as there is less competition with native blood flow [11]. Overall, our in-hospital mortality 46% which is similar to previously reported mortality rates seen in all ECMO cases [12]. Previously reported studies suggested that VA-ECMO and VV-ECMO have similar mortality rates, as well as distribution of complications to the central nervous system [13]; however, more recent studies have suggested that with improved technological advances, VV-ECMO may provide a survival advantage to certain populations [14-18]. Further larger studies are needed to delineate this and may provide a suitable alternative in those patients who do not require cardiac support.

One fact that remains is that over the past four decades the application and technology behind ECMO have changed dramatically in the face of a potentially sicker overall cohort of patients [19]. Advancements have included changes to the cannula, pumps, oxygenators, circuit configuration, and use of anticoagulation [14]. For example, according to the ELSO 2011 survey, the use of silicone membrane oxygenators continues to decline, the use of centrifugal pumps continues to increase and ECMO personnel continues to be comprised of multidisciplinary teams [20]. With those changes in technology and essentially stable mortality rates, there have been additional concerns around certain modalities. According to Thiagarajan, et al. (2013), there appeared to be an increased odd of complications related to centrifugal pumps [21]. While we did not see an overall increased mortality and percentage of complications in our patients who were exposed to centrifugal pumps, with the exception of hemorrhagic hemolysis, continued research is needed.  

The events leading to cannulation are often the most important factors related to mortality and morbidity cited in VA-ECMO studies; for example, some studies have suggested that the presence of E-CPR, irrespective of the underlying diagnosis, carries a much higher mortality rate and associated morbidities [22]. In our cohort of infants, the underlying diagnosis did still play a role in the overall mortality. Those patients with congenital diaphragmatic hernias (CDH), pulmonary failure including respiratory distress syndrome, and cardiac failure with associated shock having the highest odds of in-hospital mortality. According to a recent Cochrane review (2010), their meta-analysis on ECMO showed a potential survival benefit in four trials consisting of mature infants with severe, but potentially reversible respiratory failure [23]. All four studies showed a clear survival benefit when excluding patients with a diagnosis of CDH. The only sub-cohort whose evidence was not as clear-cut for a survival benefit were CDH patients [23]. This is similar to other studies that have stated that optimal treatment for respiratory distress, persistent pulmonary hypertension, and pulmonary hypoplasia with associated vasculature hypoplasia (CDH) remains to be defined [24-26]. Congruently, our patients with a diagnosis of CDH and pulmonary failure including respiratory distress syndrome had the highest odds of mortality despite advances in technology and intensive care. Thiagarajan, et al. (2007) also reported that E-CPR saved approximately 48% who would have otherwise died [27]. Their study also showed that those neonates with both underlying cardiac and respiratory failure performed better than their counterparts with pediatric respiratory failure [27]. Juxtaposed to their study, we did not find this same association; those where E-CPR was utilized, as well as those patients with pulmonary failure and cardiac failure, had the highest odds of in-hospital mortality.

In addition to mortality and the effect of underlying etiologies, we did observe a wide array of morbidities incurred in this cohort; albeit, the timing of these comorbidities in relation to cannulation to VA-ECMO  is not able to be determined from this registry data. There was a predominance of renal-related morbidities, including the use of hemofiltration and dialysis as being independently associated with mortality. These results are similar to results from Askenazi, et al. (2011) who have reported on the relationship between the role of fluid overload and acute kidney injury as independent predictors of morbidity and mortality in cohorts of patients primarily on VA-ECMO [9]. Acute kidney injury (AKI) may be an independent marker of mortality in of itself [28-32]; however, in our cohort, this association did not remain significant in multivariate analysis. Yet, this lack of association must be tempered by the following:  serum creatinine is often not the optimum or best measure to detect AKI or predict which patients will progress to fulminant kidney failure requiring renal supportive therapy [33]. The other confounder present is that indications for renal supportive therapy (RST) during ECMO are varied and need to be standardized in both clinical practice and semantics [34]. For example, it is unclear whether the data from each center represents true convection methods for RST (hemofiltration (HF), hemodiafiltration (HDF), ultrafiltration (UF)) or if there is some overlap between aforementioned entities and hemodialysis.

As inferred by other contemporary studies, complications occurring following the institution of VA-ECMO also incur an increased mortality [14, 35-36]. Therefore, it is not surprising that 92% of those who died in our study had experienced only one type of complication (p< 0.001) and that, along with renal complications, there was also an increased mortality associated with neurological sequelae given the physiological effects of VA-ECMO [37]. Interestingly enough, inotropes and cardiac adjuncts did not portend increased risk of mortality in this specific cohort of patients as it has in previous studies [38]. We also found that more aggressive ventilatory support, in the form of HFOV and the use of surfactants, as well as vasodilators pre-cannulation appeared to have an impact on mortality; however, this must be tempered as there is a wide variety of clinical results and center practices as it relates to pre-ECMO support [19, 39-46].

## Conclusions

We conclude that this cohort of infants experienced diverse systemic sequelae and their mortality is high (46%). Predicting morbidity and mortality in these complex patients is a constant challenge for the treatment team and is often used to assist in the counseling of their families in continuing such therapies. Adjunctive measures may need to be tempered or tailored to individual patients based on their underlying diagnosis and projected overall outcomes.
